# Clinic-based SAMBA-II vs centralized laboratory viral load assays among HIV-1 infected children, adolescents and young adults in rural Zimbabwe: A randomized controlled trial

**DOI:** 10.1371/journal.pone.0281279

**Published:** 2023-02-14

**Authors:** Vinie Kouamou, Rhoderick Machekano, Tichaona Mapangisana, Caroline Maposhere, Reggie Mutetwa, Justen Manasa, Tinei Shamu, Kathy McCarty, Shungu Munyati, Junior Mutsvangwa, Mampedi Bogoshi, Dennis Israelski, David Katzenstein

**Affiliations:** 1 Department of Medicine, University of Zimbabwe, Harare, Zimbabwe; 2 Department of Medicine, University of Stellenbosch, Cape Town, South Africa; 3 Department of Molecular Biology, Biomedical Research and Training Institute, Harare, Zimbabwe; 4 Department of Medical Microbiology, University of Zimbabwe, Harare, Zimbabwe; 5 African Institute for Biomedical Sciences and Technology, Harare, Zimbabwe; 6 Newlands Clinic, Harare, Zimbabwe; 7 Institute of Social and Preventive Medicine, University of Bern, Bern, Switzerland; 8 Graduate School of Health Sciences, University of Bern, Bern, Switzerland; 9 Chidamoyo Christian Hospital, Karoi, Zimbabwe; 10 International Partnership for Microbicides, Pretoria, South Africa; 11 Gilead Sciences Inc, Foster City, California, United States of America; 12 Department of Medicine, Stanford University School of Medicine, Stanford, California, United States of America; PLOS: Public Library of Science, UNITED KINGDOM

## Abstract

**Background:**

In Zimbabwe, children, adolescents and young adults living with HIV (CALWH) who are on public health antiretroviral therapy (ART) have inadequate viral load (VL) suppression. We assessed whether a clinic-based VL monitoring could decrease 12-month virologic failure rates among these CALWH.

**Methods:**

The study was registered on ClinicalTrials.gov: NCT03986099. CALWH in care at Chidamoyo Christian Hospital (CCH) and 8 rural outreach sites (ROS) on long-term community-based ART were randomized (1:1) to 6 monthly VL monitoring by COBAS®Ampliprep®/Taqman48® HIV-1 at the provincial referral laboratory (PRL) as per standard of care (SOC) or by the clinic-based SAMBA II assay, Diagnostics for the Real World, at CCH. VL suppression, turn-around-time (TAT) for VL results, drug switching and drug resistance in second-line failure were assessed at 12 months.

**Results:**

Of 390 CALWH enrolled 347 (89%) completed 12 months follow-up. Median (IQR) age and ART duration were 14.1 (9.7–18.2) and 6.4 (3.7–7.9) years, respectively. Over half (57%) of the participants were female. At enrolment, 78 (20%) had VL ≥1,000 copies/ml and VL suppression of 80% was unchanged after 12 months, with no significant difference between the SOC (81%) and the clinic-based (80%) arms (p = 0.528). Median (IQR) months to confirmatory VL result at CCH vs PRL was 4.0 (2.1–4.4) vs 4.5 (3.5–6.3) respectively; p = 0.027 at 12 months. Drug switching was documented among 26/347 (7%) participants with no difference between the median (IQR) time to switch in SOC vs clinic-based arms (5.1 (3.9–10.0) months vs 4.4 (2.5–8.4) respectively; p = 0.569). Out of 24 confirmed second-line failures, only 4/19 (21%) had protease inhibitor resistance.

**Conclusion:**

In rural Zimbabwe, the clinic-based SAMBA II assay was able to provide confirmatory VL results faster than the SOC VL assay at the PRL. However, this rapid TAT did not allow for a more efficient drug switch among these CALWH.

## Introduction

The World Health Organization (WHO) recommends regular viral load (VL) monitoring for people living with HIV (PLHIV) on antiretroviral therapy (ART) [1]. In the era of early and universal treatment of HIV, VL suppression to < 1,000 copies/ml provides long-term individual clinical benefit and supports treatment as prevention [2]. Effective community treatment and monitoring used as a model of differentiated service delivery (DSD) can streamline and simplify care [3, 4]. Regular VL monitoring and changes in treatment regimens sustain virologic suppression [5]. However, in many low and middle-income countries (LMICs), particularly in rural areas, there are challenges in VL monitoring [6, 7]. These challenges include limited laboratory capacity, infrastructure, long turnaround times (TAT) and poor management of VL results [8].

Standard of care (SOC) VL monitoring with central laboratory Nucleic Acid Assay Testing (NAAT) [9, 10] in many LMICs, including Zimbabwe, is performed at a provincial referral laboratory (PRL) using high throughput automated molecular assays. However, clinic-based VL assays may help augment VL testing coverage and surmount challenges faced in rural areas in Zimbabwe. In addition, for decentralized ART programs in remote rural areas, clinic-based VL testing may offer more efficient sample transport and information networks [11, 12]. Modelling studies provide evidence for cost-effective clinic-based VL monitoring [13]. New clinic-based VL using NAAT technologies have been developed, commercialized and implemented in pilot programs in many LMICs to close the gap in access [14–17]. Clinic-based laboratory instruments provide quantitative or semi-quantitative VL at district-level laboratories or primary health care settings in 90 minutes with modest laboratory infrastructure.

We hypothesized that a clinic-based VL testing could improve care in achieving VL suppression for children and adolescents receiving care through an established community program provided in a remote rural district.

We performed a randomized trial of central laboratory compared to clinic-based VL testing in a community treatment program at Chidamoyo Christian Hospital (CCH) and rural outreach sites (ROS) in Hurungwe district in Mashonaland West province in North West, Zimbabwe. The study compared virologic outcomes, drug switching and drug resistance mutations (DRMs) among children, adolescents and young adults. The aim of this study was to reduce virologic failure rates among children, adolescents and young adults in HIV care through implementation of a package of interventions that includes digital data collection, clinic-based VL monitoring and lower cost genotyping for persistently viraemic individuals.

## Methods

### Objectives

The primary objective was to determine whether the Simplified Amplification-based Assays (SAMBA), a clinic-based VL monitoring vs SOC VL monitoring decreased 12-month virologic failure rates among children, adolescents and young adults living with HIV (CALWH) in rural Zimbabwe. The secondary outcomes included rates of lost to follow up, drug switching, TAT for VL results and DRMs among these CALWH.

### Study setting

Chidamoyo Christian Hospital (CCH) is a mission hospital which serves a dispersed rural community, providing ART to over 2,500 patients in Hurungwe district, Mashonaland West, Zimbabwe. The service delivery model is a nurse-run program providing bimonthly ART drug refills and adherence counselling at either the hospital clinic or through eight ROS, 22.5 to 47 km by poor gravel roads (mean 32.8 km) from CCH [18]. Clinic and outreach visits to refill drugs, capture vital signs and provide adherence counselling are scheduled every two months. Community health workers remind the community and patients to attend. A team from CCH including a nurse, pharmacy assistant and counsellor travel to the ROS, informal meeting points where 200–400 PLHIV receive drugs bimonthly. Children, adolescents and young adults (<25 years) who lived within 10 km of CCH were treated and monitored at CCH at bimonthly youth friendly clinics where vital signs, adherence counselling, phlebotomy and drug refills were provided by nursing and pharmacy staff.

### Ethical considerations

This study was approved by the Institutional Review Board of the Biomedical Research and Training Institute (AP143/2018) and the Medical Research Council of Zimbabwe (MRCZ/A/2269). The study was registered as community-based ART (CBART) on clinical trial.gov (NCT03986099). Guardian consent was obtained for those < 7 years. Assent was obtained from 7 to 17-year old with guardian consent and those aged ≥18 years provided written consent at enrolment into the study.

### Sample size and power considerations

We estimated the required sample based on the primary outcome of VL suppression at 48 weeks. Under usual care, viral suppression rate was about 60%. We hypothesized that the clinic-based VL testing SAMBA intervention would improve care to achieve viral suppression to 90% after 12 months. We estimated that, with a total of 356 CALWH on ART, we could detect at least a 15% increase in virologic suppression with 90% power at 0.05 significance level, assuming a 10% lost to follow up.

### Study design

This was a prospective randomized open label trial of two strategies for VL differentiated care monitoring of virologic outcome among CALWH receiving ART at 8 treatment ROS near their homes provided by CCH. Between February 2018 and July 2019, 451 CALWH (ages 3–24) who had been on ART for more than 1 year at CCH or at ROS were enrolled and randomized. This was a parallel group randomized trial where only participants were blinded and assigned (1:1) to either SAMBA or SOC VL testing. The random allocation was not concealed and allocation sequences were generated by the data team, who also assigned the participants to the interventions. All study procedures and enrolment were integrated into routine care from existing clinic and outreach site staff (clinicians/nurses) as part of routine service delivery. Nurses and clinicians recruited the participants and were not blinded to the randomization. There were no anticipated risks associated with clinic-based VL differentiated care since all study procedures were consistent with the National SOC VL. Participants were seen bimonthly either through the CBART program at ROS or at youth clinics at the CCH. We abstracted data from medical records to a structured data retrieval form. Information collected included age, gender, weight, clinical and laboratory data (VL and CD4 count), ART regimen, ART initiation and cotrimoxazole prophylaxis dates, primary caregiver and site of HIV care (CCH versus ROS). Treatment dispensed, drug switching and VL measures were captured into a MS Access database.

### Randomization

Participants enrolled in the study were assigned (1:1) to the SOC VL testing at the PRL using Roche COBAS® Ampliprep®/COBAS Taqman48® HIV-1 v 2.0 or to the intervention, a clinic-based VL assay, the SAMBA II semi-Q assay (Diagnostics for the Real World, Sunnyvale California) [19] at CCH.

### Viral load monitoring by the SOC VL testing

Whole blood was collected in ethylenediaminetetraacetic acid (EDTA) tubes and plasma was prepared by centrifugation at 1,000g for 10 mins and stored at -20°C within 6 hours of collection. In the laboratory, plasma samples for SOC testing were dispatched weekly to the PRL (200 km in Chinhoyi) and a quantitative VL result with a detection range of 20 copies/ml to 10,000,000 copies/ml [20] was returned within 4–8 weeks.

### Viral load monitoring by the clinic-based SAMBA assay VL testing

The SAMBA assay is a semi-quantitative and robust HIV diagnostic platform for VL monitoring for resource-constrained settings. The SAMBA assay with the cut off of 1000 copies/ml (above or below 1,000 copies/ml) was previously validated and implemented in Uganda and Malawi [19, 21]. The authors reported an adequate accuracy when compared to the standard laboratory based-VL measurement assay (the Roche COBAS AmpliPrep/COBAS TaqMan HIV-1 test, v2.0). The overall concordance reported for the SAMBA semi-Q was 99% (95% confidence interval [CI], 93.8 to 99.9%) [19].

Similarly, whole blood was collected in EDTA tubes and plasma was prepared by centrifugation at 1,000g for 10 mins and stored at -20°C within 6 hours of collection prior to VL testing by the SAMBA assay. These samples were thawed and assayed for 90 minutes within 3 days at CCH and VL results were available to care providers within these 3 days. However, these results were not used for immediate clinical management as the children were only seen and monitored bimonthly at the hospital.

### Management of virologic failure

The 2017 Zimbabwe treatment guidelines [22] consider VL ≥ 1,000 copies/ml as potential treatment failure. A second VL test (a confirmatory sample) is only obtained two months later at the next clinic or outreach visit. Those with two sequential VL ≥ 1,000 copies/ml results are eligible to switch regimens. Second‐line participants with consecutive VL ≥ 1,000 copies/ml require a drug resistance test to determine eligibility for third-line treatment. Adherence counselling is provided at each visit. The confirmatory TAT for VL results was calculated as the time difference between sample collection date and date at which confirmatory results were available. The confirmatory result was recorded in the clinical record. The VL results at confirmatory testing and TAT were compared between the clinic-based SAMBA assay and SOC arms.

### Drug resistance testing

HIV-1 viral ribonucleic acid (RNA) was isolated from 200μl of the stored plasma samples using a column based extraction kit, the PureLink^TM^ Mini Viral RNA/DNA Mini Kit (ThermoFisher Scientific, Carlsbad, CA, USA) in accordance with the manufacturer’s instructions. The RNA was eluted in 30ul of elution buffer (E1) and stored at -80°C when not used for reverse transcription and polymerase chain reaction (RT-PCR) immediately. For the amplification, the low-cost kit-based commercial assays for HIV-1 drug resistance from ThermoFisher were used. Briefly, this is a one-step RT-PCR protocol, followed by nested PCR, which generates an amplicon of 1197 base pairs covering all the 99 HIV-1 protease codons and the first 300 codons of the reverse transcriptase (RT) of the HIV-1 *pol* gene. All amplicons were sequenced using commercial Sanger sequencing services accessed at the Molecular Cloning Laboratories (MCLab), San Francisco, California. The chromatograms generated were assembled using Geneious software, version 8 [23] and DRMs were determined using the online Stanford HIVDB program [24].

### Statistical analysis

We categorized VL as suppressed (< 1,000 copies/ml) or unsuppressed (≥ 1,000 copies/ml). Age was categorized into 3 groups: <10 years, 10–15 years and 16–24 years. We grouped primary caregivers as both parents, single parent and other non-parent relatives. We summarized baseline characteristics using frequencies and proportions for categorical variables and medians for continuous variables stratified by intervention arms. We compared baseline characteristics between the study arms using chi-square tests for categorical variables and Wilcoxon rank-sum tests for continuous variables. We examined baseline characteristics independently associated with viral suppression at enrolment using multivariable logistic regression.

To estimate the effect of the clinic-based SAMBA assay versus SOC VL testing on 12-month viral suppression rate, analyses were based on two approaches (per protocol analysis and intention to treat (ITT) analysis). In the first approach, we included only participants with 12-month virologic outcomes as assigned to study group, with inverse probability of missingness weighting to account for imbalances in baseline characteristics due to differential missingness between study arms (weighted per protocol analysis). In the second approach we used ITT analysis in which all randomized participants were included, with those missing 12-month virologic outcomes imputed to virologic failure (ITT Missing VL—failure). In all the analyses we estimated the effect of the clinic-based SAMBA assay and 95% confidence interval using logistic regression. We further examined characteristics associated with 12-month VL suppression using multivariable logistic regression. Variables associated with VL suppression with p< 0.20 in univariable logistic regressions and clinically relevant variables were included in the multivariable model.

Confirmatory VL testing and test result TAT, drug switching and VL suppression at 12 months were estimated and compared by treatment arm using chi-square tests or Wilcoxon rank-sum tests as appropriate. All statistical analyses were performed using Stata 15.1 (College Station, Tx).

## Results

### Participant characteristics at baseline

**Fig 1:**
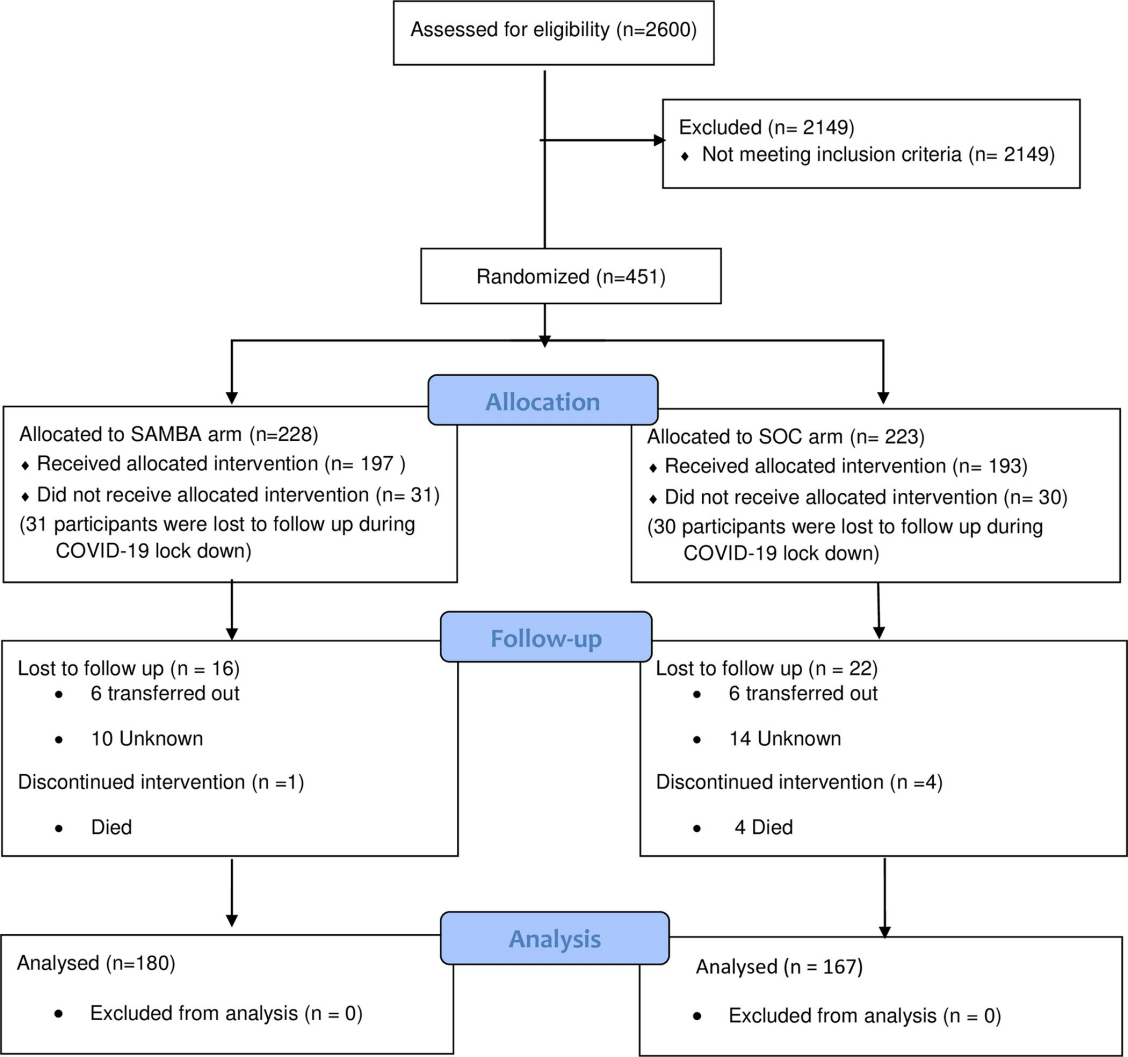
Consort flowchart for the randomized controlled trial.

A total of 451 participants deemed eligible were randomized, but the last 61 enrolled were lost to follow up during COVID-19 lock-down. Thus, a total of 390 CALWH were randomized and followed up (Fig 1). Eighty percent of the participants (312/390) were suppressed (VL< 1,000 copies/ml) at enrolment. Out of the 390 CALWH enrolled, 104/210 (49.5%) were assigned to the clinic-based SAMBA assay testing at CCH compared to 93/180 (51.7%) at ROS. There was no significant difference in all baseline characteristics in the clinic-based SAMBA assay and SOC arms. The median (IQR) age, CD4 and duration on ART was 14.1 (9.7–18.2) years, 662 (446–886) cells/uL and 6.4 (3.7–7.9) years respectively (Table 1).

**Table 1:** Participant characteristics at baseline.

Variables	Category	Overall N = 390	SAMBA assay arm N = 197	SOC arm N = 193
HIV care site	Chidamoyo	210(54%)	104(53%)	106(55%)
	Outreach clinics	180(46%)	93(47%)	87(45%)
Gender	Female	223(57%)	120(61%)	103(53%)
	Male	167(43%)	77(39%)	90(47%)
Regimens	NNRTI	237(61%)	122(62%)	115(60%)
	PI	153(39%)	75(38%)	78(40%)
CD4 (cells/uL)	<200	15(7%)	10(9%)	5(5%)
	≥200	203(93%)	98(91%)	105(95%)
	Median (IQR)	662(446–886)	633(411–890)	671(502–874)
Age (years)	<10	103(27%)	47(24%)	56(29%)
	10 to 15	108(28%)	55(29%)	53(28%)
	16 to 24	172(45%)	91(47%)	81(43%)
	Median (IQR)	14(10–18)	15(10–18)	14(10–18)
Baseline VL (copies/ml)	<1,000	312(80%)	159(81%)	153(79%)
	≥ 1,000	78(20%)	38(19%)	40(21%)
ART duration (years)	Median (IQR)	6.4(3.7–7.9)	6.3(3.5–7.9)	6.6(4.0–8.0)

SOC = Standard of care, ART = Antiretroviral therapy, PI = Protease inhibitor, NNRTI = Non-nucleotide reverse transcriptase inhibitor, VL = Viral load, IQR = Interquartile range. Chi-squared test, Fishers exact and Wilcoxon rank sum tests were used to establish relationships among variables.

### Viral load suppression at baseline

The overall VL suppression at baseline was 80%, and among CALWH tested with the Roche assay, 18% had low-level viremia (VL>20 and <1000 copies/ml). Interestingly, a significant difference was observed between baseline VL suppression and site of HIV care: significantly more participants enrolled at ROS (85%) were suppressed compared to participants from the CCH (76%) (p = 0.018). Children and adolescents on PI-based second-line regimens were less likely to be suppressed compared to those on NNRTI-based first-line regimens (73% vs 84%, respectively, p = 0.006). In addition, immune-compromised children and adolescents (CD4 <200 cells/μL) were less likely to be suppressed compared to those with a CD4 of greater than 200 cells/μL (p = 0.001).

### Viral load suppression at 12 months

**Fig 2:**
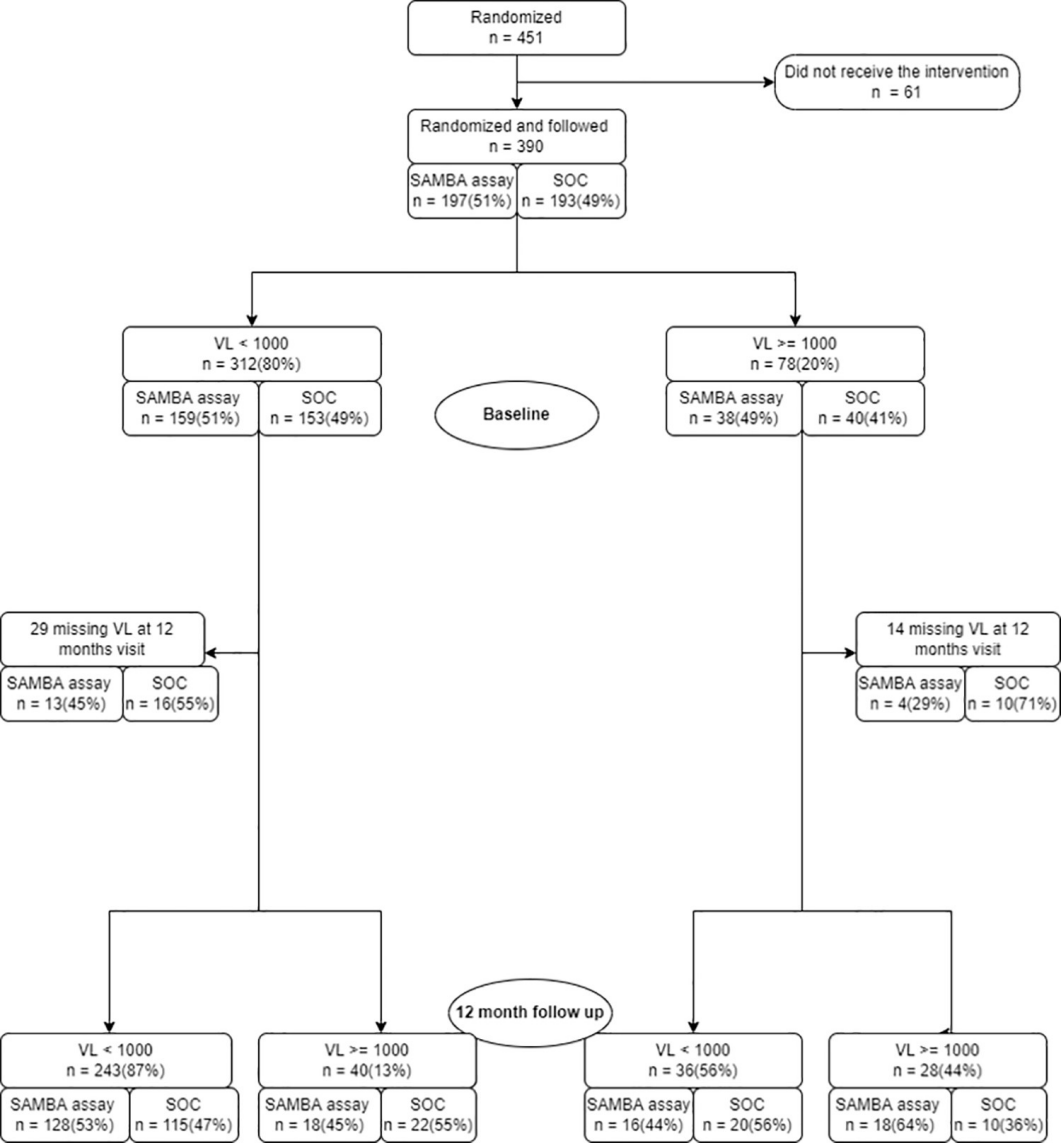
Viral load suppression cascade.

A total of 347/390 (89%) participants were followed up for 12 months. Of the 43 participants who were not tested at 12 months, 24 (56%) were lost to follow up, 14 (32%) transferred to other programs and 5 (12%) died. Among participants with 12-month virologic outcomes, 114 (80%) in the clinic-based SAMBA assay arm were suppressed compared to 135 (81%) in the SOC arm (Fig 2). Based on the ITT analysis approach, there was no significant difference in virologic suppression between the clinic-based SAMBA assay versus SOC arm (Table 2). Additionally, there was no difference in proportion missing 12 months VL between the two groups (the clinic-based SAMBA assay, 48/228 [21%] vs SOC 56/223 [25%], P = 0.306).

**Table 2:** Effect of the clinic-based SAMBA assay on viral load suppression compared to SOC.

	Weighted per-protocol analysis (n = 347)				ITT missing VL = failure (n = 451)			
	Viral load (copies/ml)				Viral load (copies/ml)			
	<1000	≥1000	OR [95% CI]	P-value	<1000	≥1000	OR [95% CI]	P-value
SOC	135(81%)	32(19%)	-	-	135(61%)	88(39%)	-	-
SAMBA assay	144(80%)	36(20%)	1.28 (0.60–2.75)	0.528	144(63%)	84(37%)	0.89 (0.61–1.31)	0.567

SOC = Standard of care, ITT = Intention to treat, OR = Odds ratio, VL = Viral load, CI = Confidence interval. In both models, we estimated the effect of the clinic-based SAMBA assay and 95% confidence interval using logistic regression.

Of the 347/390 (90%) who remained in care, 279/347 (80%) were virally suppressed. There was no significant difference in VL suppression between the SOC 135/167 (81%) and the clinic-based SAMBA assay 144/180 (80%) arms. Nineteen percent of the CALWH tested with the Roche assay had low-level viremia (VL>20 and <1000 copies/ml). Among the 78 CALWH who were unsuppressed at baseline, 14 did not have 12 month VL. Of the 64 CALWH (30 SOC and 34 SAMBA) with 12 month VL, 36 (56%) were virally suppressed. In the SOC arm, 20/30 (67%) achieved VL suppression at 12 months compared to 16/34 (47%) in the clinic-based SAMBA arm (p = 0.115).

Age group and baseline VL were independently associated with VL suppression at 12 months adjusting for gender, HIV care center, and time on regimen (Table 3). Adolescents (31%) were less likely than children under 10 years (21%) to be suppressed. Moreover, immunosuppressed CALWH with CD4<200 cells/μL (6%) were less likely to be suppressed compared to CALWH with CD4>200 (94%). Unsuppressed CALWH at entry (18%) were less likely to be suppressed compared to suppressed CALWH (82%).

**Table 3:** Characteristics associated with viral load suppression at 12 months.

Variable	Category	Overall N = 347	VL (copies/ml) at 12 months		Unadjusted OR (95% CI)	Adjusted OR (95% CI)	p-value for adjusted OR
			<1,000 n = 279	≥1,000 n = 68			
Arm	SOC	167(48%)	135(81%)	32(19%)	1	1	-
	SAMBA assay	180(52%)	144(80%)	36(20%)	1.05(0.62–1.79)	0.96 (0.54–1.70)	0.885
HIV care sites	Chidamoyo	185(53%)	146(79%)	39(21%)	1	1	-
	Outreach clinics	162(47%)	133(82%)	29(18%)	1.23 (0.72–2.09)	0.89 (0.49–1.60)	0.688
Gender	Female	194(56%)	157(81%)	37(19%)	1	1	-
	Male	153(44%)	122(80%)	31(20%)	0.93 (0.54–1.58)	0.91 (0.51–1.63)	0.762
Regimen	NNRTI	211(61%)	171(61%)	40(59%)	1		
	PI	136(39%)	108(39%)	28(28%)	0.90 (0.53–1.55)		
Baseline CD4 (cells/uL)	<200	11(6%)	6(55%)	5(45%)	1	-	-
	≥200	185(9%)	156(8%)	29(16%)	4.48 (1.28–15.67)		
Age (years)	<10	95(28%)	86(91%)	9(9%)	1	1	-
	10 to 15	104(31%)	77(74%)	27(26%)	0.30 (0.13–0.67)	0.27 (0.11–0.68)	0.005*
	16 to 24	142(42%)	111(78%)	31(22%)	0.37 (0.17–0.83)	0.39 (0.17–0.93)	0.034*
Baseline VL	<1000	283(82%)	243(86%)	40(14%)	1	1	-
	≥1000	64(18%)	36(56%)	28(44%)	0.21 (0.12–0.38)	0.20 (0.11–0.38)	<0.001*
ART duration (years)	Median (IQR)	6.5 (3.9–8.0)	6.4 (3.7–7.9)	7.3 (5.2–8.3)	0.87 (0.78–0.97)	0.90 (0.80–1.02)	0.098

SOC = Standard of care, ART = Antiretroviral therapy, PI = Protease inhibitor, NNRTI = Non-nucleotide reverse transcriptase inhibitor, VL = Viral load, OR = Odds ratio, IQR = Interquartile range, CI = Confidence interval, * = P-value statistically significant. Univariate and multivariable logistic regressions were used to establish risk factors for viral load suppression at 12 months follow up visit.

### Confirmatory viral load testing and test result turn-around-time

Confirmatory VL testing was performed after a VL result ≥ 1,000 copies/ml at an average of approximately 3 months after 6- and 12-month visits. The TAT for confirmatory VL test results was significantly shorter by the clinic-based SAMBA VL testing at CCH compared to SOC at PRL (median (IQR), 2.7(1.4–2.9) vs 3.5(2.5–5.8) months [p = 0.004] at 6 months and 4(2.1–4.4) vs 4.5(3.5–6.3) months at 12 months, respectively [p = 0.027]).

### Regimen switching

Of the 78 study participants with virological failure (VF) at enrolment, 26 (33%) switched regimens after confirmation of VL ≥1,000 copies/ml. At enrolment, 37/237 (16%) of NNRTI-based first-line ART recipients had a VL ≥1,000 copies/ml and 21/237 (9%) of the participants who were on NNRTI-based ART were switched to PI-based second-line ART. In contrast, of the participants on a PI-based second-line regimen, 41/153 (27%) had confirmed VF, but only 5/153 (3%) switched to integrase strand transfer inhibitor (InSTI)-based third-line ART. The median (IQR) time to switch regimens from the first VL ≥1,000 copies/ml was 5.1 (3.9–10.0) months in SOC VL testing arm compared to 4.4 (2.5–8.4) months by the clinic-based SAMBA VL testing, (p = 0.569).

### Genotypic analysis of second-line failures

Genotypic resistance testing was carried out among 24 participants (15 participants in the SOC arm vs 9 participants in the clinic-based assay arm) persistently failing a PI-based second-line regimen: atazanavir/ritonavir in 12, lopinavir/ritonavir in 11. One participant, who did not have PI or InSTI resistance mutations, was on darunavir + raltegravir + lamivudine/tenofovir disoproxil fumarate. Altogether, DRMs were identified in 19 of 24 (79%) participants, with 87% (13/15) in the SOC arm versus 67% (6/9) in the clinic-based SAMBA assay arm (p = 0.326, Fisher’s exact test).

Major DRMs to PIs were identified in only 4/19 (21%). Resistance to NNRTIs was found in 17/19 (89%) of the participants, with G190A being the most common mutation, found in 8 (42%) participants. The most common NRTI mutation was M184V in 12/19 (63%). Dual class resistance to NRTIs and NNRTIs was detected in 10 (53%) participants, and 3/19 (16%) had multiclass drug resistance to PIs, NRTIs, and NNRTIs. Thymidine analogue mutations were less common in these 3 participants: Two had only a T215Y mutation and the other had the M41L and T215Y mutations.

## Discussion

Children, adolescents and young adults living with HIV (CALWH) in sub-Saharan Africa have high rates of mortality and morbidity due to disproportionately lower rates of VL suppression, reduced adherence and low rates of switching to second and third-line regimens [25, 26]. New VL testing platforms are bringing NAATs to the clinic-based assays to provide more rapid VL testing on demand [14]. Here we pragmatically evaluated the implementation of VL testing among CALWH on ART in rural Zimbabwe to identify and confirm VF and switch drug regimens. After a year of focused VL testing, 319/347 (80%) had viral suppression at 12 months in this vulnerable population.

Clinic-based assays and point of care (POC) VL testing may result in more efficient and rapid drug switching. A recent study by Nicholas, et al (2019) in rural clinics in Malawi demonstrated that POC testing with SAMBA, reduced TAT for VL results and time to drug switching [21]. The authors also reported that same day POC VL results at decentralized district clinics led to switching after 6.9 months compared to 9.7 months with the district hospital with central laboratory testing. Here, we observed a significant difference in TAT for VL results between SAMBA-II VL testing and the PRL SOC central laboratory testing with drug switching after a median of 4.4 months from VF. However, the rapidity of getting the VL results by the clinic-based SAMBA assay did not make any difference to clinical care as drug switching was not different between the two arms, constrained by the bimonthly visit schedules and confirmatory VL on a second test after a VL ≥1,000 copies/ml plus the 2–3 months adherence counselling. The service delivery model at the hospital is a nurse-run program where drug refills, examination of vital signs, phlebotomy and adherence counselling are only scheduled every two months. Furthermore, drug switch at the hospital was done as per the National HIV guidelines (after adherence counselling and failure of a confirmatory VL).

Although the OR point estimate for the weighted model is slightly greater than 1, and less than 1 in the ITT missing VL = failure model, the overall result is the same, there is no significant difference in VL suppression between the two arms. The main reason for the different direction of the effect of SAMBA compared to SOC might be because of the assumption of treating all missing VL as failure. Since SOC has a higher proportion of missing 12 month VL compared to SAMBA (13% vs. 9% per protocol and 25% vs 21% per ITT), the suppression rate in the SOC arm is almost the same compared to the SAMBA arm (81% vs. 80% respectively). However, this assumption does not effectively change the conclusions of the study. Studies of assays of lower level VL thresholds (>20–999 copies/ml) suggest that the WHO-recommended ≥1,000 copies/ml threshold may underestimate virologic failure [27, 28]. Here, analyzing the SOC arm tested with the Roche assay, up to 18% of CALWH had VL measures > 20 and <1000 copies/ml at baseline. Moreover, recent models have shown that switching based on a single VL result at the WHO threshold of ≥ 1,000 copies [29] could be cost-effective.

We found that the SAMBA-II semi quantitative assay is well suited to identifying VL failure at the ≥ 1,000 copies/ml threshold and was simple to manage in a rural hospital setting with limited power, internet access and telecommunication. New clinic-based assays and POC technologies offering rapid quantitative POC VL at lower thresholds [14] may further improve care to achieve VL suppression in rural resource limited settings. Additional studies of POC VL testing using the Xpert (Cepheid, Paulshof, SA) confirmatory test and drug switching are underway in adults in South Africa, Nigeria and Haiti [30–32]. However, SAMBA and these new POC technologies are instrument-, reagent-, and supply chain-dependent and require quality assurance and training for laboratory and nursing staff [33].

Access to InSTI-based-third-line treatment has been restricted by the requirement for genotyping and the demonstration of major PI resistance mutations as prerequisites for consideration of InSTI-based, third-line treatment and darunavir-based regimens. Here we found that only 4/24 (17%) second-line failures had major PI resistance despite persistent VF as described in urban Zimbabwe [34, 35]. Although not statistically significant, the risk of acquiring resistance was slightly higher in the SOC arm (87%) vs the clinic-based assay arm (67%). This may be due to smaller sample size and lack of power to draw conclusions on these rates. However, the rapid identification of patients with VF may reduce the rate of emergence of resistance as well as the risk of failure of subsequent ART regimens.

Care at Chidamoyo provides long-term retention and support through community-based counselling and ART [18]. Interestingly, VL testing in the community demonstrated significantly better VL suppression among those receiving care at ROS compared to those attending the clinics at CCH. However, this difference narrowed with systematic VL testing over the 12 months of the study to 82% vs 79% (p = 0.80). The inadequate adherence, reduced tolerability and selection of DRMs associated with current NNRTI- and PI-based regimens among children and adolescents [34, 36, 37] may be alleviated by the implementation of the fixed dose combination of tenofovir disoproxil fumarate/ lamivudine/dolutegravir [38, 39].

Strengths of our study include the use of relatively large sample of participant data from a largely decentralized HIV programme in Zimbabwe and that the clinic-based SAMBA VL testing was successfully performed by the clinic medical team at the Chidamoyo clinic. Sustaining long-term ART and VL testing in rural Zimbabwe in CALWH requires innovative approaches to DSD to improve adherence, retention in care and faster drug switch [40].

The major limitation of our study includes the fact that, the clinic-based SAMBA VL testing did not help in rapidly facilitating adherence counselling and faster regimen switch after identifying VF, due to the bimonthly visit programme at CCH. Another limitation includes the high lost to follow up resulting in only 89% (347/390) of VL tests being used in the final analysis, possibly underestimating VL testing coverage. The power of the study to be able to determine a difference between the clinic-based SAMBA assay and SOC VL testing was also limited due to relatively good rates of viral suppression at baseline and limited duration of follow-up.

## Conclusions and recommendations

We found that in rural Zimbabwe, the clinic-based SAMBA II assay for VL testing can achieve high testing coverage in rural Zimbabwe and reduced TAT for VL results. However, the rapid TAT did not allow for a more efficient drug switch constrained by the bimonthly visit schedules. Despite access to VL monitoring, VL suppression among these CALWH did not reach 90%. A study-adapted visit schedule is recommended in future trial. This study design would help ascertain the effectiveness of clinic-based VL testing in reducing TAT for VL results and drug switch and consequently improving VL suppression among CALWH in rural Zimbabwe.

VL testing remains important to identify those with adherence and access issues to mitigate the selection of drug resistance. Monitoring VL using a mixture of clinic-based assays and central laboratory testing, adapted to HIV programs serving vulnerable populations, will simplify ART with the widespread use of more effective drug regimens to end the HIV pandemic.

## Supporting information

S1 Appendix Study protocol. (DOCX)

S2 Appendix Consort checklist. (DOC)

S1 Data (XLSX)

## List of abbreviations

1. ART: Antiretroviral Therapy
2. CALWH: Children, Adolescents and Young Adults living with HIV
3. VL: Viral Load
4. CCH: Chidamoyo Christian Hospital
5. ROS: Rural Outreach Sites
6. PRL: Provincial Referral Laboratory
8. TAT: Turn Around Time
9. IQR: Interquartile Range
10. PLHIV: People Living with HIV
11. DSD: Differentiated Service Delivery
12. LMICS: Low-and-middle-Income Countries
13. SOC: Standard of Care
14. NAAT: Nucleic Acid Assay Testing
15. DRM: Drug Resistance Mutations
16. CBART: Community Based ART
17. EDTA: Ethylenediaminetetraacetic Acid
18. RNA: Ribonucleic Acid
19. RT- PCR: Reverse Transcription and Polymerase Chain Reaction
20. RT: Reverse Transcriptase
21. MCLAB: Molecular Cloning Laboratory
22. IIT: Intention to Treat
23. MRCZ: Medical Research Council of Zimbabwe
24. LTFU: Lost to Follow up
25. PI: Protease Inhibitor
26. NNRTI: Non Nucleos(t)ide Reverse Transcriptase Inhibitor
27. OR: Odds Ratio
28. CI: Confidence Interval
29. InSTI: Integrase Strand Transfer Inhibitor
30. TLD: Tenofovir disoproxil fumarate/ Lamivudine/Dolutegravir
