# Supplementary material for: Clinic-based SAMBA-II vs centralized laboratory viral load assays among HIV-1 infected children, adolescents and young adults in rural Zimbabwe: A randomized controlled trial
Source: PLoS One. 2023 Feb 14;18(2):e0281279. doi: 10.1371/journal.pone.0281279 (PMC9928130; doi:10.1371/journal.pone.0281279)
Supplement: S1 Appendix — (DOCX) [file pone.0281279.s001.docx]

**Title: Community Based Virus Load Differentiated Care in Rural Africa**

***Short Title: Community Based ART (CBART)***

***A randomized, open label trial of two strategies for VLDC monitoring of virologic outcome in a rural community based treatment program in Zimbabwe.***

Principal Investigator: Dr. Shungu Munyati

Biomedical Research and Training Institute

Protocol Version Number: 3.4 August 8, 2018

Table of Contents

[2 Investigators and their roles 3](#_Toc473911153)

[3 Protocol Summary 4](#_Toc473911154)

[3.1 Background 5](#_Toc473911155)

[3.2 Justification for Study 6](#_Toc473911156)

[Treatment Outcomes among adolescents with HIV in Africa 6](#_Toc473911157)

[Viral load monitoring 7](#_Toc473911158)

[4 Specific Objectives and design 9](#_Toc473911159)

[4.1 Objectives and Measures 9](#_Toc473911160)

[4.2 Primary Objective 9](#_Toc473911161)

[4.3 Secondary Objectives 9](#_Toc473911162)

[Methods 10](#_Toc473911163)

[4.4 Study Setting 10](#_Toc473911164)

[4.5 Study population 11](#_Toc473911165)

[4.5.1 PARTICIPANT WITHDRAWAL 15](#_Toc473911166)

[5 DRUG RESISTANCE GENOTYPING. 16](#_Toc473911167)

[6 Data Management and Analysis 16](#_Toc473911168)

[6.1 Data Analysis Plan 17](#_Toc473911169)

[7 Statistical analysis 18](#_Toc473911170)

[8 Ethical considerations 18](#_Toc473911171)

[8.1 Consent process 19](#_Toc473911172)

[9 Risks: 19](#_Toc473911173)

[9.1 The risks associated with participating in the study are considered to be moderate, and are comparable to risks associated with clinical care. 19](#_Toc473911174)

[10 Benefits 19](#_Toc473911175)

[11 Confidentiality 19](#_Toc473911176)

[STUDY MONITORING 19](#_Toc473911177)

[21. Timeline 20](#_Toc473911178)

[11.1 STUDY DISCONTINUATION **Error! Bookmark not defined.**](#_Toc473911179)

[12 References 24](#_Toc473911180)

[13 Appendices Error! Bookmark not defined.](#_Toc473911181)

# Investigators and their roles

Dr. David Katzenstein, MD (Professor of Medicine Stanford, emeritus) is the Director of the Molecular Diagnostics Laboratory at the Biomedical and Research Training Institute of Zimbabwe (BRTI). He will be responsible for oversight of the laboratory, clinical informatics and study design and assumes responsibility for biosafety, human subjects and ethical conduct of research.

Shungu Munyati, PhD is the Director-General of the BRTI and the Interim Chairperson of the Zimbabwe Public Health Association. Dr. Munyati has managed multiple HIV and TB evaluation studies, particularly in epidemiology and social medicine. Her research work with BRTI, Ministry of Health & Child Care and UZCHS is focused on health service delivery, particularly for HIV-affected children in society.

Justen Manasa, PhD (Global Health Equity Scholar Stanford/UC Berkeley) has recently completed a post-doctoral fellowship in drug resistance and phylogenetics with Robert Shafer and Benjamin Pinsky at Stanford. He has completed studies of drug resistance and phylogenetics at the Africa Centre for his PhD from UKZN in virology (2015).

Junior Mutsvangwa, PhD and BRTI Director of Labororatory logistics and Monitoring and Evaluation officer at BRTI.

Kathy McCarty, RN. Clinical Nurse manager directs the ART program and CBART at Chidamoyo Christian Hospital.

**Clinical Site**:

Chidamoyo Christian Mission Hospital

Karoi, Mashonaland West, Zimbabwe

Kathie McCarty, RN, PhD (Director of Operations)

Dr. Munodawafa, MBChB, (Medical Officer) Major Merrick, (Hospital Administrator)

**Laboratories**

Newlands Clinic Dr. Cleophas Chimbetete, MBChB, Paser Phd Cand

Tinei Shamu, MPhil Laboratory director

BRTI 10 Seagrave Rd., Avondale Harare

Justen Manasa, PhD (Medicine)

BRTI Molecular Diagnostics Lab:

Junior Mutsvangwa,

Vinie Kouamou

Alfred Makura, B.S. Mphil Candidate

Bhavini Varyani, MSc

**Consultants**: Benjamin Pinsky, MD PhD, (Stanford) and Anat Rosenthal, PhD (Ben Gurion University Medical Anthropology). Catherine Maposhere,

Statistical support Rhoderick Machekano, PhD (Pediatric AIDS Foundation)

# Protocol Summary

In 2014 UNAIDS set ambitious goals to achieve 90% virologic suppression rates among individuals on ART. In Zimbabwe <5% of HIV infected individuals received HIV viral load (VL) testing in 2016 and treatment failure rates among children and adolescents range between 35-42% with high rates of drug resistance. Scalable strategies to facilitate improved VL monitoring; genotyping and drug switching for those with persistent viremia are urgently needed.

**Our primary objective is to implement “Virus load differentiated care” (VLDC) using mobile health tools to assess near Point of care (POC) vs a Standard of Care (SOC) viral load monitoring to mitigate virologic failure of HIV-infected** *among children, adolescents and young adults.*

The proposal is an open label randomized trial among HIV infected children and young adults receiving ART at 8 treatment outreach sites near their homes provided by Chidamoyo Mission Hospital. We will implement VL testing at “near point of care” using the SAMBA to evaluate the safety, clinical and virologic outcomes of near POC monitoring of virus load at the Chidamoyo Christian Hospital in Mashonaland West Zimbabwe. We hypothesize that our proposed package of care will result in a decrease in virologic failure, increase virologic suppression and prevent drug resistance in this key population in a rural ART treatment program. Process and cost data will be collected for subsequent cost-analysis.

All eligible ART patients served by Chidamoyo Christian Hospital (< 25 years of age n = 700) will be asked to participate in the study at regular visits when they receive ART. The Ministry of Health is rolling out a new (2017) standard of care (SOC) including semi-annual virus load (Roche Cobas VL) through the Provincial Laboratory at Chinhoyi (100 km from Chidamoyo).

Up to 600 HIV infected children and young adults on ART will be randomized (1:1) to either SOC (300) or a near POC (300) VLDC monitoring. SOC VL among 300 is performed by Roche COBAS at the Provincial Hospital Chinhoyi and the results returned to the hospital within 4 weeks. Those randomized to near POC (n=300) will be tested with the SAMBA and results are available within 3 days. Follow-up repeat testing for HIV RNA > 1,000 copies/ml is offered using the same virologic monitoring system at the next drug/clinic visit within 3 months.

The hypothesis is that viral load monitoring and potentially genotyping to sustain suppression to < 1,000 copies/ml will reduce treatment failure to < 15% from the ~ 33% virologic failure rate currently recorded among young people on therapy. Secondary endpoints include the rate of drug switching and the evaluation and prevention of drug resistance. The study will enroll up to 600 children 5-10 years, adolescents 10-19 and young adults 20-25 years, providing data that will guide strategies for management of children, adolescents and young adults surviving on ART.

***Primary Objective:*** *To determine if implementation of point of care virus load differentiated care (POC virus load), targeted genotyping and mHealth tools will result in improved virologic suppression among children, adolescents and young adults (<25 years) on ART.*

*Sample size*

**The primary study endpoint** is viral load suppression at 48 weeks among PLWHA < 25 years old, using VLDC implemented as near POC compared to SOC semi- annual virus load testing. We will enroll up to 600 young PLWHA from eight communities and the Chidamoyo Hospital Clinic as a rolling prospective cohort from the more than 700 individuals < 25 years of age currently receiving ART. We hypothesize that an intervention package of digitized data, local immediate POC Virus load by SAMBA and genotype will result in > 90% virologic suppression after 1 year. The estimated minimum sample size to detect at least a 15% increase in virologic suppression with 90% power, at significance level $\alpha=0.05$ assuming 10% loss to follow up rate (LTFU) rate is 356 PLWHA on ART.

**Secondary endpoints:**

**1. rate of switching from 1^st^ to 2^nd^ line.**

**2. frequency of DRM among 1st and 2^nd^ line virologic failures.**

**3. Eligibility for 3^rd^ line ART**

**3. genotype of first and second line failures.**

**4. The frequency of Hepatitis B virus infection (HBSag).**

**In collaboration with Ben Gurion University Global Health, we will perform an ethnographic survey over the course of the primary study. The objective is to develop formative research to understand the individual and community variation in suppression rate and drug switching including differences in virologic failure and adherence by age, gender, rural outreach site, orphan-hood/caregiver and socio-economic status. (see appendix for ethnographic study).**

**Introduction**

## Background

With universal test and treat, a growing number of HIV infected children are diagnosed, treated and surviving on ART. Long-term adherence and retention in care of HIV-infected children, adolescents and young adults pose significant challenges to health care systems, particularly those which have limited laboratory and monitoring capacity. This is especially critical to HIV care in resource-limited settings where virologic suppression and preserving therapeutic options is key to UNAIDS 90-90-90 goals for HIV diagnosis, ART care and **virologic suppression**. A key population, children, adolescents and young adults in care have virologic failure rates of > 25% far below the goal of 90% viral suppression. Limited capacity for viral load (VL) monitoring results in delayed detection of treatment failure, deferral of changes in drug regimens and accumulation of drug resistance mutations with the potential for emergence and transmission of multi-drug and class resistant (MDR) virus.

Virus Load Differentiated Care (VLDC) requires high throughput virus load testing on sophisticated platforms and laboratory infrastructure which is only just becoming available through the MOH. In Zimbabwe in 2015, less than 5% of HIV infected individuals have received an HIV viral load test (standard of care Roche Cobas CAP/CTM).

## As a POC test the Simple AMplification Based Assay (SAMBA) has been approved for VL monitoring. Standard of Care (SOC) virus load testing, annually for lower risk and semi annually for high risk subjects, including children and adolescents has been rolled out and is available from the provincial medical centre in Chinhoyi. The results are physically printed and sent within 1-2 month to the Chidamoyo hospital.

In contrast SAMBA can be performed at Chidamoyo Hospital in 90 minutes. These laboratory results, provided in near real time (within 3 days) by the hospital laboratory as digitized VL information will be communicated to the clinic, providers and pharmacy on android devices. Although VL monitoring detects early virologic failure, ART management, adherence counseling and a repeat virus load are integral to maintaining virologic suppression. Community based ART includes caregivers, family and community health workers who supplement the usual clinic work-flow minimizing costs and enhancing support for children and adolescents.

Interventions are only effective in sustained viral suppression in those with virus that is susceptible to their ART regimen. The cost of genotyping has limited it’s use in clinical decision making in sub-Saharan Africa. Lower-cost genotyping assays identify key mutations that arise following exposure to first and second line regimens can guide certain drug switches and substitutions, adherence interventions and therapeutic decisions. A genotype can impact clinical care by differentiating adolescents and children who have discontinued ART from those with intermittent or inconsistent use. We hypothesize that a package of interventions focused on VL monitoring coupled with the option for lower cost genotyping for those with persistent viremia, will result in significant improvements in virologic suppression rates among children and adolescents receiving HIV care within public programs including remote rural sites in Zimbabwe. We will test this hypothesis in 8 rural outreach sites in Mashonaland West and at the ART clinic at the district hospital/clinic and pharmacy at Chidamoyo where regular visits for ART maintenance are scheduled every 3 months.

## Justification for Study

Treatment Outcomes among adolescents with HIV in Africa

There are over 2.6 million HIV infected children globally^1^. The majority of adult and pediatric infections are in sub-Saharan Africa where more than 60% of HIV infected individuals are living in rural communities with minimal investments in health care infrastructure. In rural settings, community service delivery, including provision of drugs and monitoring at the point of care, supports survival of HIV infected individuals and prevents the transmission of drug resistant virus. However, from 2005 to 2012, HIV-related deaths among children and adolescents increased by 50%, while the global number of HIV-related deaths fell by 30%^2^. This increase in adolescent HIV-related deaths is due to limited access to testing, treatment, monitoring and care services, limited support for retention and adherence to ART. Despite increasing access to ART, high virologic failure rates, stigma and non-disclosure result in declining CD4 counts and progression to AIDS, even among children and adolescent in care in urban settings.^3^


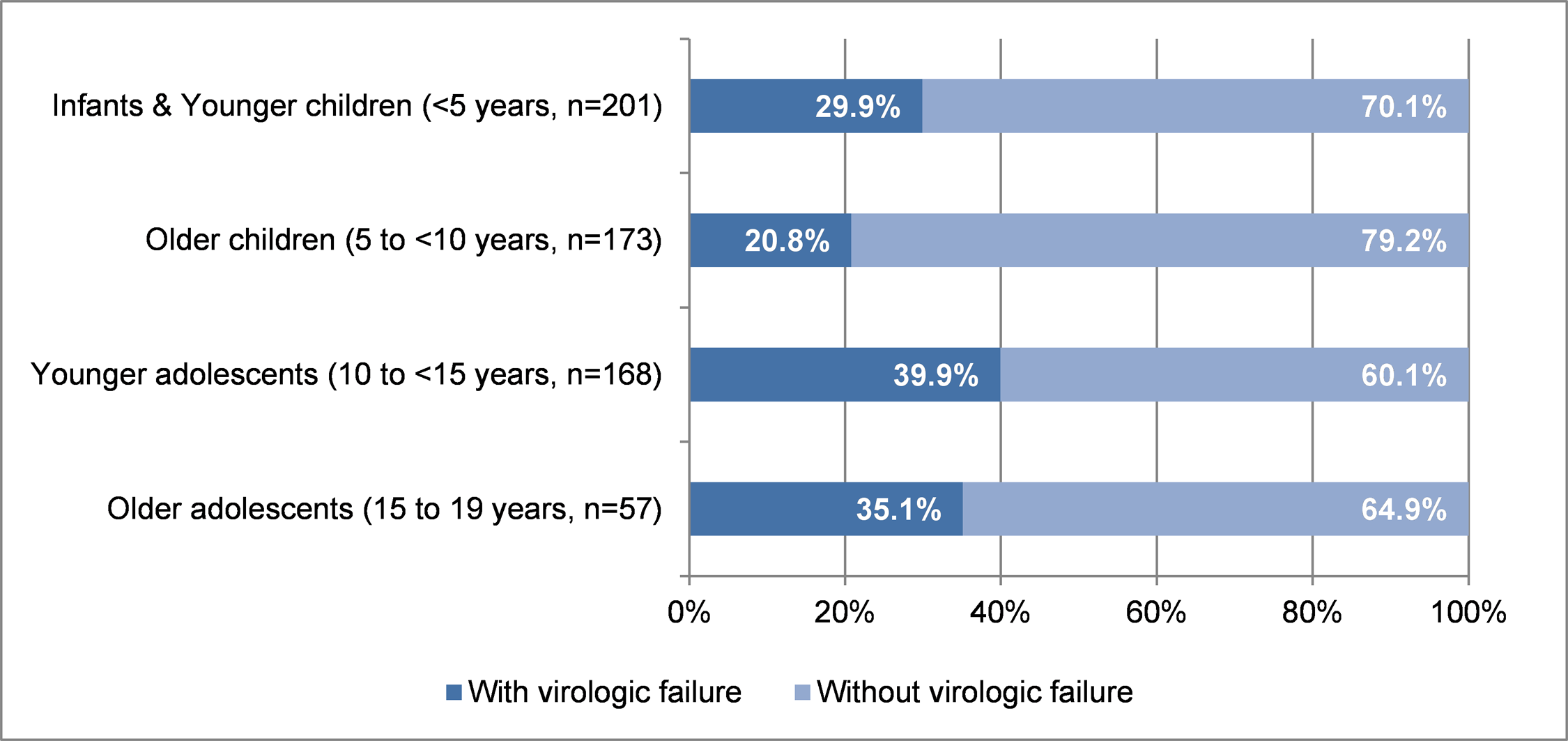
Figure 1. High Virologic Failure rates among HIV infected children and adolescents in care.^3^ Virologic failure has been linked to poor adherence, lack of HIV status disclosure and psychosocial challenges. Cost-effective, culturally appropriate, community-based solutions are required to reverse these trends. However limited access to viral load monitoring further delays detection of treatment failure in much of sub-Saharan Africa. **High virologic failure rates hinder the preservation of first and second-line therapies and limit future therapeutic options.**

**Preliminary results**. A study (SEKA) in Hurungwe at CCH conducted in 2016 found that 100/300 children and adolescents on ART receiving CBART at 8 outreach sites were viremic (>1,000 copies/ml) indicating a rate of virologic failure of 33%. An addition (10%) had low level viremia (LLV) between 60 and 1,000 copies/ml. Similarly in a recent evaluation of 132 adolescents and young adults in Harare, 39% of those on second-line therapy had VL>1000 copies/ml.

### Viral load monitoring

In 2013, the WHO introduced routine viral load monitoring into guidelines for the management of individuals living with HIV in resource and capacity limited settings. Recently Virus Load differentiated care (VLDC) as presented by Phillips et al ^4^ has been shown to be more cost-effective in management of ART. By 2016, only a few countries in sub-Saharan Africa were providing access to routine viral load monitoring**.** HIV-1 viral load monitoring of patients on ART has been limited by costs which extend beyond reagents and includes the skilled human resources, sample collection, transportation, processing, and infrastructure support and equipment maintenance. Several POC diagnostic platforms and assays that have recently been approved that can achieve viral quantification from plasma^5^. However, in Zimbabwe, in 2016 only 5% of the patients in the national ART program had received viral load testing (MOHCC, Zimbabwe). **Near POC SAMBA for routine viral load monitoring will simplify specimen collection and processing, leveraging existing equipment and networks for sample transport to increase access to VLDC.**

**Drug Resistance Monitoring**.

Virus load testing is an effective tool to identify patients who are not responding to ART and is recommended annually and if feasible in key populations, every 6 months by the WHO in low and middle income countries (LMIC)^6^. Viremia on ART, despite intensified counseling and good adherence, indicates acquired drug resistance and requires switching to second line ART to achieve viral suppression and reduce HIV transmission to partners. Optimal selection of second line ART is best guided by genotyping using Sanger sequencing, but access, cost, turnaround time, and laboratory and transport infrastructure limits availability in LMIC. In-house resistance genotyping assays can greatly reduce cost and increase access to drug resistance monitoring^8^. We will test the hypothesis that low cost genotyping in the context of public health ART will optimize outcomes. Genotyping will guide appropriate switching to second line or third line regimens, and reduce the emergence, duration and potential transmission of drug resistant mutations.

**Lower cost genotyping**.

We have developed an in-country lower cost VL through sequencing by the mail. This assay may be performed on Dried blood spots (DBS), which can be stored for long periods of time and transported with minimal impact on stability and sample quality^17-19^. There may be advantages in the collection of DBS samples particularly in infants and children where obtaining sufficient blood by phlebotomy is often difficult. Data from pilot data in Zimbabwe shows that genotyping to monitor for infants and children and PLWHA living in remote or rural sites can be achieved using DBS as the analyte for genotyping.

**Community Based ART for children, adolescents and young adults at Chidamoyo Christian Hospital.**  Health care and ART have been administered to a population of 200,000 by the Chidamoyo Christian Mission Hospital. The CCMH has developed sustainable health care delivery systems, delivering drug to 8 decentralized communities (insert map) serving nearly 2,000 ART recipients and provides drug to another 2,000 PLWHA who receive ART medications at the hospital. The unique outreach program for Drug delivery is accomplished in a one day visit, repeated every two or three months (8 - 12 weeks) as community outreach in which 3 health care workers (Pharm Tech, Community educator and Nurse) deliver ART to 150-250 individuals. The visit includes clinical evaluation and referral/treatment as necessary. We propose to add low cost VL using SOC (Roche) or near POC (SAMBA) virus load by obtaining blood at community or clinic visits. Among those with a confirmed VL > 1,000 copies/ml second-line therapy is provided based on Public Health MOHCW ART guidelines. Among those on 2^nd^ line, transport of samples to Harare, with a turnaround time for results of 4 weeks to determines eligibility for 3^rd^ line (DTG/DRV) therapy. A robust digital text-based communication system will be used to transmit the VL information to the provider, clinic and if desired the patient as well as the MOH, PMD offices as a standardized form entered on tablets and transmitted via 3G to the portable cloud mini servers.

Community-based ART (CBART) can succeed in drug treatment and monitoring using new technology that includes digital health records, compact single tablet regimens (STR), lower-cost point of care diagnostics for virus load differentiated care using real-time actionable information. At rural outreach treatment sites with limited access, real time point of care laboratory results will lead to more timely patient management. This will provide a lower cost solution to VLDC supported by Chidamoyo Christian Mission Hospital and a Fogarty Information, Communication and Technology (ICT) program at the University of Zimbabwe and the BRTI, a G-11 Fogarty training award (TW010320-01). Cepheid, which has recently achieved approval of a point of care virus load assay, is providing tools for the integration of data to evaluate scalable and cost-effective virus load differentiated care to impact patient management and prevent drug resistance.

# Specific Objectives and design

**Aim 1:** To determine if implementation of a package of care that includes near POC SAMBA, viral load monitoring decreases 12-month virologic failure rates among HIV-infected children, adolescents and young adults.

**Aim 2:** To adapt and integrate a digital medical record using android Tablets to collect data in the field and a low cost server to aggregate and consolidate data at the clinic, hospital and district level to identify actionable virologic failure, confirm virologic failure and switch treatment to second or third-line therapy to maintain virologic suppression.

**Aim 3:** To implement low-cost genotyping for the detection of drug resistance to determine eligibility for the next treatment line appropriate to the resistance mutations identified.

## Objectives and Measures

The aim of this study is to reduce virologic failure rates among children, adolescents and young adults in HIV care through implementation of a package of interventions that includes digital data collection, near point of care VL monitoring and lower cost genotyping for persistently viremic individuals. The objectives are:

## Primary Objective

1. To determine if near POC Virus load monitoring vs SOC VL monitoring decreases 12-month virologic failure rates among HIV infected children, adolescents and young adults.

## Secondary Objectives

1. To identify rates of loss to follow-up, regimen changes and drug substitution, morbidities and toxicities among children, adolescents and young adults LWHA in the context of CBART in a rural district on first and second line treatments.
2. To determine the prevalence of drug resistance mutations and multi-drug resistant viruses (MDR) among children and adolescents on ART receiving care in a largely rural public health settings.
3. To determine if there is variation in effectiveness of POC VL between the rural outreach sites and the district hospital, and if so, how the outreach population site specific characteristics such as size of HIV+ population and the organizational structure, impact the effectiveness and delivery of the intervention.
4. To identify the frequency of Hepatitis B infection (HBSag, HBcore) in children, adolescents and young adults on ART.

## Methods

**Study Design**: Prospective randomized open label study in 8 rural out-reach sites and Chidamoyo Hospital of VLDC comparing SOC and nPOC monitoring of ART.

**Study Population and Setting**

Children adolescents and young adults (3 to 25 years) receiving HIV care are eligible for enrolment.

**Virologic failure rates in Zimbabwe**

In a survey of adolescents in care in 2015, virologic failure rates were 30% among children (0-10 years) and **32% among adolescents in Mashonaland West**. In Matabeleland North a Roche CAP/CTM platform was installed in Hwange in 2015 and 1200 viral load tests were performed. **Virologic failure among those below 21 years was 43%. Review of the ZIMPHIA study findings extended these to young adults. See Figure 2.**

**
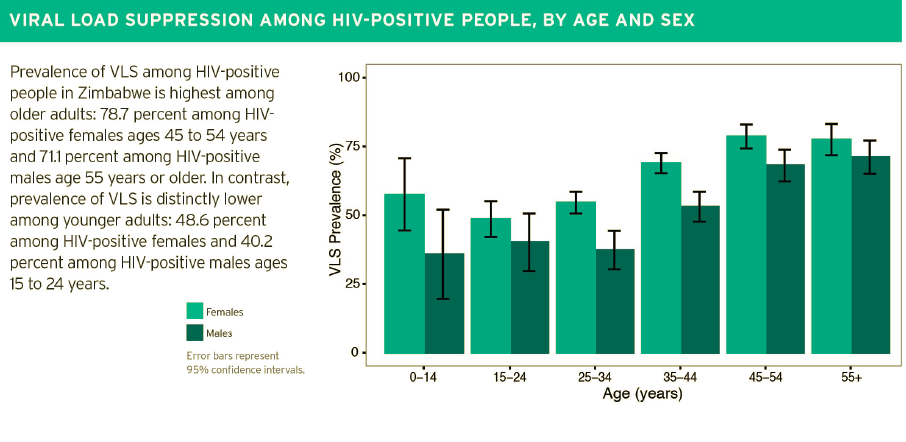
**

**Figure 2: Viral load suppression among HIV-positive people, by age and sex in Zimbbabwe**

.

Finally in 2016 a cross sectional survey at Chidamoyo of 300 children and adolescents found a 33% rate of virologic failure and a study of adolescents in Harare on second line therapy demonstrated nearly 50% VF^9^.

## Study Setting

**Study Site**

.

The Chidamoyo Mission in Hurungwe district in Northwest Zimbabwe provides diagnostic and treatment services to a rural population with more than 4,000 PLWHA receiving HIV treatment. Nearly 1600 of these PLWHA receive care within 2 km of their rural homes through a visiting team of nurses/community workers who deliver treatment every three months.

<https://www.buzzfeed.com/joncohen/want-to-end-aids-this-village-in-zimbabwe-knows-how?utm_term=.acGrWKBBR#.symV6gEEw>.

Community-based ART programs can succeed in drug treatment and monitoring using new technology that includes digital health records, compact single tablet regimens (STR) and lower-cost point of care diagnostics for virus load differentiated care. This provides real-time actionable information towards a critical biomedical outcome with scalable mHealth tools. We propose to use applications for point of care data collection (Open Data Kit) and open Medical Record Systems (open MRS) on the PortableCloud ([portable cloud](http://www.portablecloud.net)), a low cost robust server and communication system to capture data from multiple tablets and smart-phones. At the 8 rural outreach treatment sites, serving PLWHA on treatment, ART is delivered 3 monthly and samples are obtained for VLDC. However, with limited access to broadband, tools are needed to integrate real time point of care laboratory results, public health reporting and effect timely patient management in these PLWHA who are rarely seen in clinic. The capture of drug dispensing data, logging samples and providing results via tablet will provide a low cost solution. Training and implementation of tablet data capture is supported by a collaboration between Chidamoyo Christian Mission Hospital and a Fogarty ICT program at the University of Zimbabwe and the BRTI, a G-11 Fogarty training award (TW010320-01). Cepheid, which has recently gained approval of a point of care virus load assay is providing new tools for VLDC which require the integration of data to evaluate scalable and cost-effective virus load differentiated care to impact patient management and prevent drug resistance.

## Study population

**Recruitment of Patients**

Children and young adults < 25 (n > 700) who were registered in 2016 in HIV care and on ART will be eligible. At rural outreach sites (n=8) where all PLWHA on drugs are reviewed every two or three months by the visiting team from the Chidamoyo ART program.

**Inclusion criteria: PLWHA will be enrolled in age based strata;**

children (5-9) , adolescents (11-19) and young adults (20-25 years) who have been receiving ART for at least 6 months from the CCH.

**Exclusion Criteria:**  Participant and caregiver unable to give consent and assent.

**Intervention/treatment**

All study procedures are integrated into routine care from existing clinic and outreach site staff as part of routine service delivery will deliver the study interventions.

**Randomization**. Prior to the scheduled prescription drug delivery visit to each of the 8 outreach CBART sites, up to 20 individuals < 25 years of age currently receiving ART for > 6 months will be randomly assigned (1:1) to standard of care (SOC) or near point of care (POC) monitoring at the upcoming visit. After enrollment, subsequent VL measures are obtained using either Central laboratory Roche or near point of Care SAMBA performed at CCH.

**Study procedures**: At the baseline/entry visit eligible patients will be consented/assented to participate. Those who agree to randomization will be provided with an explanation of the aims of the study. There are no anticipated risks associated with bi-annual VLDC since all study procedures are consistent with the national Standard of Care. Participants enrolled will receive an HIV VL at baseline (SOC-Roche and / or near point of care (SAMBA). Results of SOC VL testing will be communicated to the clinic from the MoH provincial laboratory in Chinhoyi. Those randomized to nPOC (SAMBA) will continue to have their blood tested by local SAMBA to inform clinical management including targeted virus load, confirmatory testing after adherence counseling, re-testing after switches in therapy and repeat HIV VL. Those randomized to SOC will be tested using SOC (Roche) at Chinhoyi provincial hospital.

* DBS are obtained from all samples and may be used exclusively for infants and as an option for children who would prefer to avoid phlebotomy, or are a “hard stick”.

Data obtained at the entry clinic visit will include enrollment and consent or assent and include the signature of the caregiver for those < 18 years of age. The demographics and location history, detailed review of the patient chart/record for baseline HIV specific history, duration of previous and current ART exposure and history of relevant opportunistic infections (particularly TB) will be entered into the digital medical record. At the baseline visit the adolescent and the caregiver will be consented and educated on significance of and interpretation of a VL test. Repeat HIV VL will be conducted at 6 months. All DBS samples will be transported to Harare where viral nucleic acids will be extracted from DBS cards and stored for later processes such as genotyping if required.

To determine the efficacy of Virus load differentiated care detailed information on patient outcomes at 12-months Roche plasma virus load test (SOC) will be collected. Data collected at each study visit will include detailed interval history, information on changes in ART regimen, new opportunistic infections, morbidity hospitalizations and mortality. All viral load results; baseline, 6 and 12-month samples will be reported back to the clinic site electronically by SMS/email and with printed results. An HIV genotype will be performed on:

1. Baseline/entry, 6 month and 12-month samples for all individuals who have an HIV VL>1000 copies/mL .
2. Baseline samples for all individuals who switched ART during the course of the study period.
3. Samples obtained for VLDC where the provider requests a genotype.

Study Schema. Participants who are randomized to SOC or nPOC monitoring undergo routine clinical evaluation at three month intervals by clinic nurses as per standard of care. At designated times (baseline, 6, and 12-months (M12) virus load testing is performed. Participants who miss the scheduled lab-draw visit at the clinic or out-reach site will have consented to home visits to collect a sample for VL monitoring. As the number of patients enrolled in each outreach cluster (rural treatment site) is small (no more than 80 patients per cluster) blood draws per site will occur on the routine visit when the team comes to distribute drugs. A repeat VL at the next visit at 3-months will be conducted in individuals who have a viral load result that is >1000 copies/mL.

**Monitoring ART recipients using point of care virus load**.

Consenting / assenting eligible ART recipients (those ≤25 years) at each community visit are enrolled in the study and those in nPOC arm are provided VL using the SAMBA virus load test at Chidamoyo Hospital. The laboratory capacity with 4 SAMBA units is ~ 20 tests per day.

The results are obtained from each rural community and the hospital clinic in the week following the visit and can be communicated directly to the patients’ records in preparation for the next visit to the patient, provider and the rural site HCW.

Viral load results will be made available to the study sites within 1 week of sample collection. Nursing staff will receive a tablet that will be used to convey patient viral load results by sms and email. Mobile phone penetration rates are high in Zimbabwe and all sites have access to cellphone coverage. Sites with cell-phone coverage will be encouraged to print and save a paper record of the laboratory results. Printed results will be delivered to sites that do not have access to cellphone coverage within 10-14 days of sample collection. Detailed results including information on management plan will be provided to the clinicians only.

Results for patients with VL>1000 copies/ml will be transmitted to CCH and the community health workers with information/suggestion on the next management step. This would include pre-prepared standardized text messages such as: “Patient *(coded number),* HIV VL result is *x copies/mL*, and meets the threshold for concern for virologic failure. Please contact the patient and ask them to present to the clinic within the next 4 weeks.”

Existing mechanisms to provide community based education, support and patient tracing, e.g. peer counselors or environmental health technicians (EHTs), will be used contact patients who are failing therapy. Participants with an HIV VL>1000 copies who attend the next outreach visit after counseling and adherence reinforcement will be asked to repeat VL and DBS collection. For infants, nurses will collect blood on DBS cards. Existing mechanisms for transportation of whole blood and DBS samples to the laboratories (nPOC and SOC) are well established. Clinic staff will be instructed to ‘bleep’ the study administrative center (the BRTI) if the site phone has insufficient funds and the study will electronically top-up air-time. The study phones will be used solely for the purposes of conveying information regarding VL testing and adverse clinical events to and from the field, clinic and laboratory.

The repeat VL result will be conveyed to the clinic by sms/email or paper. For patients who have shown a good response to therapy (>1,000 c/ml in repeat VL after) a standard message such as “Patient *(coded number),* VL was *x copies/mL* on DD/MM/YY and repeat VL on DD/MM/YY was *x* *copies/mL*. The viral load is showing the anticipated response in the setting of improved adherence. Repeat viral load should be scheduled at the 6-month follow up visit”. For patients who are not responding to therapy, a reflex genotype from viral nucleic acids extracted from the most recent plasma or DBS sample will be performed. A message informing the clinician of the results will suggest appropriate options/regimens. A typical message may be as follows: “Patient (*coded number)* VL was *x* *copies/mL* on DD/ MM/YY and repeat VL on DD/MM/YY was *y* *copies/mL*. The viral load is not improving on therapy. If the clinic physicians request a resistance test, remnant sample or DBS will be used to perform a genotype and a message will be sent to the clinic “A genotype test was done and the following mutations were detected (*insert mutations)*. Based on this and previous ART drug exposure we recommend that the patient be switched to (*insert new ART regimen)*. Please do not hesitate to contact the study team if you have any additional questions”.

All study participants receive a repeat viral load at 6 months. Participants who are viremic at 6-months will undergo the same procedures as detailed above including repeat viral load monitoring after receipt of results, adherence support, and repeat viral load with genotyping if requested in the setting of persistent viremia. All DBS cards will be transported to the laboratory in Harare for storage. In addition to HIV viral load, HIV genotype will be done on all individuals who are viremic at the 12-month follow-up. There will be three laboratory hubs for the study; a Roche CAP/CTM at the Chinhoyi MOH provincial lab. The BRTI – AiBST Harare lab will serve as the central genotyping lab hub for the study. VL results above 1000 copies/mL will be reported to the study medical officer. If the VL is a repeat VL>1000 copies/mL reflex genotyping will be initiated. The laboratory director Justin Manasa, PhD will review results and patient history and determine the appropriate message to be sent to the clinic and the patient. The study research assistant will be responsible for electronically disseminating the results and the appropriate message to the clinics and patients.

Study visit schedule.

All enrolled patients are seen every 3 months for drug treatment. After consenting at enrolment a virus load is obtained. If > 1,000, the provider is notified and a repeat virus load is performed within weeks, or at the next visit (in 3 months). Confirmed VL > 1,000 will lead to a genotype and if drug resistance to the current regimen a recommendation for drug switching.

**Statistical analysis**

*Sample size*

The primary study endpoint is viral load suppression for 48 weeks/12 months among PLWHA < 25 years old after the mHealth-POC intervention compared to virologic suppression rates with mHealth-standard of care monitoring. We will enroll 500 young PLWHA from eight communities and Chidamoyo Hospital; 300 receiving the SOC and 300 in the proposed POC VL intervention. Current suppression rate is < 60%. We hypothesize that the mhealth interventions; digitized ePMS data and local POC Virus load will achieve > 90% virologic suppression after 12 months. The estimated minimum sample size to detect at least a 15% increase in virologic suppression with 90% power, at significance level $\alpha=0.05$ assuming 10% loss to follow up rate (LTFU) rate is 356 PLWHA on ART. With an accrual rate of 60 patients per month, we expect to enroll a total of 600 PLWHA on ART.

*Analysis plan*

We will use means/median for continuous variables and proportions for categorical variables to describe the study population by intervention arm. The primary objective of this study is to estimate the suppression rate (<1,000 copies/ml) among children and adolescents on TDF/3TC+EFV (first line maintenance regimen), the rate of switching to second line ATV/r + 3TC/AZT and estimate the proportion (and associated confidence intervals) who remain suppressed for 48 for weeks. We will perform exploratory subgroup analyses, estimating suppression rates for subgroups defined by gender, age groups and other characteristics. Similarly, we will summarize outcomes on adverse events and resistance using proportions and associated confidence intervals.

L**oss to follow-up**

We will define lost to follow-up as failing to collect drug after 1 month of scheduled receipt/visit. To minimize potential bias associated with loss to follow-up, every effort will be made to retain an enrolled participant in the study for 12-months. Optimal participant retention procedures will be in place to ensure that the loss to follow-up rate is less than 10%. These procedures will include

- Explanation of all study procedures including study visits at the time of consenting and enrollment as well as at each study visit.
- Collection of detailed locator information at the screening visit as well as review at each study visit (residential address, telephone contact details of participant, and next of kin)
- Text message reminders to participants regarding study related appointments.

Immediate follow-up following missed visits. Participants who miss scheduled visits will be contacted by text message to remind them to reschedule a visit. If this fails, 3 telephone call attempts will be made. If these are unsuccessful, a home visit will be arranged. This will be done to identify reasons for loss to follow-up and/or allow the participant the option to voluntarily withdraw from the study.

### PARTICIPANT WITHDRAWAL

Participants may voluntarily withdraw from the study for any reason at any time. Participants may also be withdrawn if the study sponsor, government, regulatory authorities, Data Safety and Monitoring Board (DSMB), or Institutional Review Boards (IRBs) choose to terminate the study prior to its planned end date. The investigator may also withdraw participants from the study in order to protect their safety and/or if they are unwilling or unable to comply with required study procedures. Reasonable effort will be made to complete a final evaluation of participants who voluntarily withdraw prior to termination of the study. Reasons for withdrawal will be recorded.

Qualitative Data Collection

Qualitative data collection will be designed to understand how VLDC is perceived and implemented. We hypothesize that a significant source of variation in outcomes for virologic suppression will be based on individual and outreach site characteristics. A questionnaire will be developed and data collected which will include data on site organization characteristics, HIV patient list size for pediatrics, adults and adolescents. During the recruitment process the outreach and clinic teams will obtain detailed information. These individuals are public health practitioners play an important role in guiding the study.

# DRUG RESISTANCE GENOTYPING.

**Research design and rationale:** In Aim 3, we will evaluate the clinical efficacy of genotypic resistance testing in patients who fail first-line NNRTI, or a second-line protease-inhibitor-based regiment. Outcomes will be the profiling of prevalent resistance mutations in individuals who do not re-suppress. The Sanger technique does not detect minority variants (defined as > 2 and < 20% of the viral population). These minority variants however may be important determinants of treatment outcomes. In a subpopulation of samples we will also perform next-generation sequencing (Illumina MiSeq, San Diego, CA) to identify minority variants.

**Samples:** DBS samples will be obtained from study participants at each scheduled visit with genotyping done on those samples with persistent VL>1000 copies/mL on two consecutive occasions 1 month apart.

**Sanger sequencing** will identify specific mutations from plasma and DBS nucleic acid amplicons. **Genotypic resistance testing protocol:** After nucleic acid isolation from DBS, samples will undergo genotypic resistance testing using the CDC resistance assay: one-step RT-PCR is followed by nested PCR to amplify 1.1 kb of HIV-1 *pol*, including codons 13–99 of PR and ≥250 codons of RT. The amplicon will be resolved on a 1% agarose gel, extracted (MiniElute kit; Qiagen), and quantified using the Nanodrop. A minimum of 5ng of amplicon will be stored for subsequent evaluation of low frequency resistant variants by MiSeq NGS (see below). Six sequencing primers are used with standard Big Dye chemistry to create overlapping chromatograms, which will be edited using Geneious v9 software.

The HIVdb program (http://hivdb.stanford.edu) will be used to interpret the drug resistance profile from the nucleotide sequences and to calculate a genotypic susceptibility score (GSS) for each antiretroviral drug using well-established. A total score will be calculated for the nationally recommended second-line ART to assess the effect of observed drug resistance mutations on the predicted effectiveness of second-line ART. A second-line regimen with a GSS<2 will be defined as compromised. For quality control, unedited sequencing files will be evaluated using ReCALL software for automated base calling and clinical resistance evaluation to ensure accurate detection of low-level variants that may be overlooked during manual editing.

# Data Management and Analysis

**Sample size and power calculation**

The proposed study will focus on children, adolescents and young adults who have high underlying treatment failure rates compared with adults. Children and Adolescents have complex needs including psychosocial needs that can affect adherence to medications and subsequent clinical outcomes. Currently the absence of viral load monitoring allows for prolonged therapeutic failure, resulting in multi-class HIV drug resistance. We hypothesize that viral load monitoring and genotyping to identify drug resistance and facilitate early switch of therapy will result in improved clinical and virologic outcomes. Based on previous data we estimate virologic failure rates to be 30-35% and that this could be reduced to 15%, the failure rates among adults in routine care.

In addition, our previous data suggests that 30% of children and adolescents with virologic failure have susceptible virus, and could remain on their current regimens with appropriate adherence support. The remaining 70% have drug resistance mutations that may require therapeutic switches^7^. The cost of pre-mature switches in those with susceptible viruses includes the cost of 2^nd^-line therapy and results in introduction of more pills, with more complex schedules and unwanted side effects. On the other hand the cost of not switching therapy when needed results in prolonged failure with the risk of progression to AIDS and accumulation of increased resistance to several classes of antiretroviral drugs.

The primary endpoint of the study will be reducing virologic failure rates and achieving the suppression rates among adults on ART care in Zimbabwe (>85-90%). We hypothesize that POC virus load and genotyping will be able to achieve this level of virologic failure (< 15%) and compare this to SOC monitoring. We assume that the variation in suppression will be largely between age groups and between sites based on factors such as differences in community organizations, counseling and support within the community and the ability of guardians and parents to manage ART access and adherence among children and adolescents.

## Data Analysis Plan

*Analysis plan*

We will use means/median for continuous variables and proportions for categorical variables to describe the study population by intervention arm. The primary objective of this study is to estimate the suppression rate (<1,000 copies/ml) at 1 year among children and adolescents on TDF/3TC+EFV (first line maintenance regimen) or among those who have been switched to second line. Secondary endpoints are the rate of switching to second line ATV/r + 3TC/AZT and an estimate of the proportion (and associated confidence intervals) who remain suppressed for > 24 weeks. We will perform exploratory subgroup analyses, estimating suppression rates for subgroups defined by gender, age groups and other characteristics. Similarly, we will summarize outcomes on adverse events and resistance using proportions and associated confidence intervals.

Data management plan

All potential participants will be given a study screening number and those who assent/consent to randomized VLDC as SOC or POC testing will be provided with a study ID number including the study arm (p or s). A study log will be established that will include screening ID number, study ID number, name, ~~national ID number~~, address details of next of kin and contact telephone number. The study log will be kept separate from all other study documents and secured with access by authorized study staff and regulatory authorities only. All patient encounters and documentation will be done using the study ID number only.

Data will be collected on tablets completed by primarily research nurses. All case reporting forms will be collected from clinic sites and reviewed by the data manager for accuracy and completion. Data will entered into an electronic database that will be specifically developed for the study. The data will stored in a central repository that can be accessed by the data manager, principal investigator and co-principal investigator. In- built data quality checks will be present in the database and will be reviewed on a regular basis by the data manager with weekly reporting to the principal investigator and/or co-principal investigators. Access to completed eCRFs will only be provided to the principal investigator and co-principal investigators, coordinator, data manager and statistician only.

Data will be backed up on an external server and further backed up in the cloud. Access to all electronic versions of the data will be password protected with access granted to the principal investigator and co-principal investigator, data manager and statistician only.

Authorized representatives of the funding agency and regulatory bodies may be provided access as needed to the eCRFs and electronic database. The data will be owned by the Biomedical Research and Training institute and Chidamoyo Mission Hospital. Study information will not be released to any party without written permission and the removal of all patient identifiers.

# Statistical analysis

The proportion of children, adolescents and young adults on ART with a detectable viral load will be estimated for the two study arms. The effect of the POC monitoring will be estimated by the relative risk of failure proportions between the SOC and POC monitoring arms after completion of all enrolled and randomized subjects at 6 months and 12 months. Binomial regression models will be used to estimate the relative risk, associated standard errors of the effect and 95% confidence intervals. We will model the probability of virological failure for individual $j$, $p_{j}$, as$\log\left( p_{j} \right)= \beta_{0}+\beta_{1}X_{j}$ where $\beta_{0}$ is the average log probability of virlogic failure in the control arm, $X_{j}$ is an indicator of monitoring arm for individual $j$, and $\beta_{1}$ is the log relative risk of the intervention compared to control (a measure of the effect of the intervention). We can add additional terms (variables) to the models to adjust for any baseline imbalances. We will use the Stata 14.0 generalized linear models with the binomial family and log link options to fit the model parameters.

# Ethical considerations

The study protocol, consent form and participant education and recruitment materials will be reviewed by the BRTI Research Ethics Committee and the MRCZ.

Any changes to the protocol must be approved by local IRBs. The principal Investigator will provide a safety and progress report to the IRB on an annual basis and will include the number of study participants and outcomes, changes in study procedures, and protocols, and unanticipated problems involving risk to human subjects.

## Consent process

Those below 18 will need parental consent and assent. Those 18 and above-can provide consent. Written informed consent with the form describing study purpose, procedures, anticipated risks and benefits, will be obtained from each study participant. All informed consent procedures will occur in the preferred language of the participant. All consent forms will be written in English, translated to Shona and back-translated into English to check for accuracy. Literate participants will document their provision of informed consent by signing their informed consent forms. Non-literate participants will be asked to document their informed consent by marking their informed consent forms (e.g,with a thumbprint) in the presence of a literate third party witness. Participants will be provided with a copy of their informed consent forms.

# Risks:

## The risks associated with participating in the study are considered to be to risks associated with clinical care.

# Benefits

The primary benefit of participating in the study is to receive optimized clinical care with close laboratory monitoring by dedicated staff with experience in the management of HIV. All participants will get optimized laboratory monitoring prior to and while on ART at no additional cost to the participant. In addition, participants will inform national and regional guidelines for the management of adolescents living with HIV.

# Confidentiality

All case reporting forms, laboratory specimens, reports, and study related records will be identified by coded number to maintain confidentiality. All records will be kept in locked file cabinets within the BRTI. All computer entry and networking programs will be done with coded numbers only with limited computer access to study personnel and care providers only. Clinical information will not be released to any party without written permission, except as necessary for authorized study monitoring.

# STUDY MONITORING

The study sponsor, may conduct monitoring or auditing of study activities to ensure the scientific integrity of the study and to ensure the rights and protection of study participants.  Monitoring and auditing activities may be conducted by an authorized representatives of the sponsor (e.g., a contracted party considered to be “external”) both internal and external parties. Monitoring or auditing may be performed by means of on-site visits to the Investigator’s facilities or through other communications such as telephone calls or written correspondence.  The visits will be scheduled at mutually agreeable times, and the frequency of visits will be at the discretion of the sponsor. During the visit, any study-related materials may be reviewed and the Investigator along with study staff should be available for discussion of findings. The study may also be subject to inspection by regulatory authorities (national or foreign) as well as the International Ethics Committees/Institutional Review Boards to review compliance and regulatory requirements.

#

# Timeline

**Timeline for implementation and evaluation of SOC vs POC monitoring and virus load differentiated care among 600 CAY on ART.**

**First 300 are baseline patient registers with implementation for the second 300 in year 1**

**Year 1 2018**

1 2 3 4 5 6 7 8 9 10 11 12

**training BRTI--------------**

**1^st^ line cumulative enrollment 50 /mo* randomly assigned (1:1) to SOC or nPOC (SAMBA)**

**150 300 450 600**

**estimated 20 % VF rate commence second-line therapy Virologic failure (> 1,000 copies/ml)**

**30 60 90 180**

**Year 2 2019**

**SAMBA vs Roche VLDC capture of prescribing, virologic outcomes; sequence 2^nd^ line VF and implement 3^rd^ line**

**Activity 1 2 3 4 5 6 7 8 9 10 11 12**

**1^st^ line 120 240 360 420**

**follow-up 2^nd^ line 30 60 90 180**

**30% of 2^nd^ line , > 1,000 copies/ml**

**adherence counseling evaluate for 3^rd^ line 18 30 60**

#

# REFERENCES

**REFERENCES**

1. UNAIDS. AIDSinfo database, 2016 estimates. http://aidsinfo.unaids.org/ Accessed Feb 23, 2017.
2. UNICEF Data: Monitoring the Situation of Women and Children. Updated Jun 2016. http://data.unicef.org/topic/adolescents/hivaids-2/ Accessed Feb 23, 2017.
3. Makadzange AT, Higgins-Biddle M, Chimukangara B, Birri R, Gordon M, Mahlanza T, et al. (2015) Clinical, Virologic, Immunologic Outcomes and Emerging HIV Drug Resistance Patterns in Children and Adolescents in Public ART Care in Zimbabwe. PLoS ONE 10(12): e0144057. doi:10.1371/journal.pone.0144057
4. Andrew N Phillips and Working Group on Modelling of Antiretroviral Therapy Monitoring Strategies in Sub-Saharan Africa. Sustainable HIV treatment in Africa through viral-load-informed differentiated care Nature 528, S68-S76 (3 December 2015), DOI: 10.1038/nature16046
5. Phillips AN, Cambiano V, Nakagawa F, et al. Point-of-Care Viral Load Testing for Sub-Saharan Africa: Informing a Target Product Profile. Open Forum Infectious Diseases. 2016;3(3):ofw161. doi:10.1093/ofid/ofw161.
6. World Health Organization (WHO). Consolidated Guidelines on the use of antiretroviral drugs for treating and preventing HIV infection. Recommendations for a public health approach. Geneva, 2013.
7. Cohen J. https://www.buzzfeed.com/joncohen/want-to-end-aids-this-village-in-zimbabwe-knows-how?utm_term=.acGrWKBBR#.symV6gEEw
8. Nachega J et al. Antiretroviral therapy adherence, virologic and immunologic outcomes in adolescents compared with adults in Southern Africa. JAIDS. 2009; 51: 65-71.
9. Garcia de Olalla P et al. Impact of adherence and highly active antiretroviral therapy on survival in HIV-infected patients. JAIDS. 2002; 30: 105-110.
10. Idele P et al. Epidemiology of HIV and AIDS among adolescents: current status, inequities, and data gaps. JAIDS. 2014; 66 (suppl 2): S144-153.
11. Kasedde S, Luo C, McClure C, and Chandan U. Reducing HIV and AIDS in adolescents: opportunities and challenges. Current HIV/AIDS reports. 2013; 10: 159-168.
12. Karanja S, Mbuagbaw L, Ritvo P, Law J, Kyobutungi C, Reid G, et al. A workshop report on HIV mHealth synergy and strategy meeting to review emerging evidence-based mHealth interventions and develop a framework for scale-up of these interventions. The Pan African medical journal. 2011;10:37. PubMed PMID: 22187619. PMCID: 3240930.
13. Fraser HS, Allen C, Bailey C, Douglas G, Shin S, Blaya J. Information systems for patient follow-up and chronic management of HIV and tuberculosis: a life-saving technology in resource-poor areas. Journal of medical Internet research. 2007;9(4):e29. PubMed PMID: 17951213. PMCID: 2223184.
14. Kurth AE, Spielberg F, Cleland CM, Lambdin B, Bangsberg DR, Frick PA, et al. Computerized counseling reduces HIV-1 viral load and sexual transmission risk: findings from a randomized controlled trial. J Acquir Immune Defic Syndr. 2014 Apr 15;65(5):611-20. PubMed PMID: 24384803. PMCID: 3999203.
15. Bateman AC, Parham GP, Sahasrabuddhe VV, Mwanahamuntu MH, Kapambwe S, Katundu K, et al. Clinical performance of digital cervicography and cytology for cervical cancer screening in HIV-infected women in Lusaka, Zambia. J Acquir Immune Defic Syndr. 2014 Oct 1;67(2):212-5. PubMed PMID: 24977474. PMCID: 4162769.
16. Chi BH, Vwalika B, Killam WP, Wamalume C, Giganti MJ, Mbewe R, et al. Implementation of the Zambia electronic perinatal record system for comprehensive prenatal and delivery care. International journal of gynaecology and obstetrics: the official organ of the International Federation of Gynaecology and Obstetrics. 2011 May;113(2):131-6. PubMed PMID: 21315347. PMCID: 3071887.
17. Arya M, Kumar D, Patel S, Street RL, Jr., Giordano TP, Viswanath K. Mitigating HIV health disparities: the promise of mobile health for a patient-initiated solution. Am J Public Health. 2014 Dec;104(12):2251-5. PubMed PMID: 25322292. PMCID: 4232104.
18. WHO. END OF AIDS 2014 [Available from: http://www.unaids.org/en/resources/campaigns/World-AIDS-Day-Report-2014.
19. UNAIDS. 90 90 90 2014 [Available from: http://www.unaids.org/en/resources/campaigns/World-AIDS-Day-Report-2014.
20. Gupta RK, Jordan MR, Sultan BJ, Hill A, Davis DH, Gregson J, et al. Global trends in antiretroviral resistance in treatment-naive individuals with HIV after rollout of antiretroviral treatment in resource-limited settings: a global collaborative study and meta-regression analysis. Lancet. 2012 Oct 6;380(9849):1250-8. PubMed PMID: 22828485. PMCID: 3790969.
21. Kotaki T, Khairunisa SQ, Witaningrum AM, M MQY, Sukartiningrum SD, Diansyah MN, et al. HIV-1 transmitted drug resistance mutations among antiretroviral therapy-Naïve individuals in Surabaya, Indonesia. AIDS Research and Therapy. 2015;12(1):1-7.
22. WHO. Global Action Plan on HIV Drug Resistance 2017-2021 2016 [Available from: http://www.who.int/hiv/drugresistance/hivdr_darft_gap.pdf?ua=1.
23. Oldenburg CE, Barnighausen T, Tanser F, Iwuji CC, De Gruttola V, Seage GR, 3rd, et al. Antiretroviral Therapy to Prevent HIV Acquisition in Serodiscordant Couples in a Hyperendemic Community in Rural South Africa. Clin Infect Dis. 2016 Aug 15;63(4):548-54. PubMed PMID: 27208044. PMCID: 4967606.
24. Catalani C, Philbrick W, Fraser H, Mechael P, Israelski DM. mHealth for HIV Treatment & Prevention: A Systematic Review of the Literature. The open AIDS journal. 2013;7:17-41. PubMed PMID: 24133558. PMCID: 3795408.
25. Oluoch T, Santas X, Kwaro D, Were M, Biondich P, Bailey C, et al. The effect of electronic medical record-based clinical decision support on HIV care in resource-constrained settings: a systematic review. International journal of medical informatics. 2012 Oct;81(10):e83-92. PubMed PMID: 22921485.
26. Makadzange AT, Higgins-Biddle M, Chimukangara B, Birri R, Gordon M, Mahlanza T, et al. Clinical, Virologic, Immunologic Outcomes and Emerging HIV Drug Resistance Patterns in Children and Adolescents in Public ART Care in Zimbabwe. PLoS One. 2015;10(12):e0144057. PubMed PMID: 26658814. PMCID: 4678607.Centers for Disease Control and Prevention (CDC). Compendium of Evidence-Based Interventions and Best Practices for HIV Prevention. 2015. http://www.cdc.gov/hiv/prevention/research/compendium/ma/

# Additional References

# 1. UNAIDS. AIDSinfo database, 2016 estimates. <http://aidsinfo.unaids.org/> Accessed Feb 23, 2017.

# 2. UNICEF Data: Monitoring the Situation of Women and Children. Updated Jun 2016. <http://data.unicef.org/topic/adolescents/hivaids-2/> Accessed Feb 23, 2017.

# 3. Chimukangara B, Varyani B, Shamu T, Mutsvangwa J, Manasa J, White E, Chimbetete C, Luethy R, Katzenstein D. HIV drug resistance testing among patients failing second line antiretroviral therapy. Comparison of in-house and commercial sequencing. J Virol Methods. 2017 May;243:151-157. doi: 10.1016/j.jviromet.2016.11.010. PMID: 27894862

#### [Associations Between Antiretroviral Treatment and Avascular Bone Necrosis: The Swiss HIV Cohort Study](https://academic.oup.com/ofid/article/4/4/ofx177/4090954/Associations-Between-Antiretroviral-Treatment-and?searchresult=1)

[Cornelia Bayard](https://academic.oup.com/ofid/search-results?f_Authors=Cornelia+Bayard), [Bruno Ledergerber](https://academic.oup.com/ofid/search-results?f_Authors=Bruno+Ledergerber), [Markus Flepp](https://academic.oup.com/ofid/search-results?f_Authors=Markus+Flepp), [Thanh Lecompte](https://academic.oup.com/ofid/search-results?f_Authors=Thanh+Lecompte), [Estelle Moulin](https://academic.oup.com/ofid/search-results?f_Authors=Estelle+Moulin) ...

Open Forum Infectious Diseases, Volume 4, Issue 4, 1 October 2017, ofx177, <https://doi.org/10.1093/ofid/ofx177>

Chimukangara B, Manasa J, Mitchell R, Nyabadza G, **Katzenstein** D, Masimirembwa C.

[**Community Based Antiretroviral Treatment in Rural Zimbabwe.**](https://www.ncbi.nlm.nih.gov/pubmed/28899102) **AIDS Res Human Retroviruses. 2017 Sep 13. [Epub ahead of print] PMID:28899102**
